# Microenvironment complexity and matrix stiffness regulate breast cancer cell activity in a 3D in vitro model

**DOI:** 10.1038/srep35367

**Published:** 2016-10-13

**Authors:** Marta Cavo, Marco Fato, Leonardo Peñuela, Francesco Beltrame, Roberto Raiteri, Silvia Scaglione

**Affiliations:** 1 National Council of Research (CNR) – IEIIT Institute, Genoa, 16149, Italy; 2 University of Genoa – Department of Biophysical and Electronic Engineering (DIBRIS), Genoa, 16145, Italy; 3 National Council of Research (CNR) – IBF Institute, Genoa, 16149, Italy

## Abstract

Three-dimensional (3D) cell cultures represent fundamental tools for the comprehension of cellular phenomena both in normal and in pathological conditions. In particular, mechanical and chemical stimuli play a relevant role on cell fate, cancer onset and malignant evolution. Here, we use mechanically-tuned alginate hydrogels to study the role of substrate elasticity on breast adenocarcinoma cell activity. The hydrogel elastic modulus (E) was measured via atomic force microscopy (AFM) and a remarkable range (150–4000 kPa) was obtained. A breast cancer cell line, MCF-7, was seeded within the 3D gels, on standard Petri and alginate-coated dishes (2D controls). Cells showed dramatic morphological differences when cultured in 3D versus 2D, exhibiting a flat shape in both 2D conditions, while maintaining a circular, spheroid-organized (cluster) conformation within the gels, similar to those *in vivo*. Moreover, we observed a strict correlation between cell viability and substrate elasticity; in particular, the number of MCF-7 cells decreased constantly with increasing hydrogel elasticity. Remarkably, the highest cellular proliferation rate, associated with the formation of cell clusters, occurred at two weeks only in the softest hydrogels (E = 150–200 kPa), highlighting the need to adopt more realistic and a priori defined models for *in vitro* cancer studies.

A great deal of experimental evidence has shown that mechanical stimuli from the cell microenvironment play a key role in affecting several types of cell behaviour, both in healthy and in pathological conditions^1^,^2^,^3^. In particular, cells sense their microenvironment via trans-membrane proteins and consequently regulate several physiological processes such as migration, proliferation, differentiation, morphology and gene expression, as well as the response to drugs^4^,^5^,^6^.

*In vivo*, cells are embedded within a complex three-dimensional gel – the Extracellular Matrix (ECM) – that provides mechanical support while directing cellular behaviour^1^,^7^. Interestingly, more and more literature shows that the ECM also plays a relevant role in the onset of a considerable number of diseases: for example, it has been shown that ECM biomechanical properties directly influence and are influenced by the progression of neoplastic disease^8^,^9^, while, during metastatic invasion, ECM rigidity can affect the motility of carcinoma cells^10^,^11^.

However, the vast majority of *in vitro* studies, particularly in cancer research, fail to fully replicate the *in vivo* situation, since they are carried out in two dimensions (2D), such as in standard Petri dishes. Extensive research has confirmed that 2D experiments are subject to various limitations, such as dissimilarities in cell adhesion and migration or in cytoskeletal organization, along with a poor analysis of complex cell-substrate interactions^12^,^13^,^14^,^15^. Consequently, 2D *in vitro* models are often associated with contradictory results, typical of transposing new medical and anticancer compounds from the bench to the bedside^16^. In particular, the lack of reliability seems to be associated with the following main aspects: cell source (e.g. phenotype selection), model dimensionality and microenvironment complexity^17^.

Another main issue in cancer biology regards the use of animal models. Human tumour cells are typically injected into nude animals to form tumour masses and metastases^17^. However, the safety and efficacy of animal studies cannot generally be transferred to human trials: the average rate of successful correspondence between animal models and clinical trials is nowadays less than 8%^18^,^19^. Moreover, *in vivo* animal models do not allow direct investigation of specific microenvironmental cues or their influence on cellular evolution, and present well-known ethical and cost-related limits^18^.

A wide range of new 3D *in vitro* models is emerging to better mimic the physiological human context and, at the same time, to reduce animal experiments. In cancer research, these clinically relevant *in vitro* models could help in understanding tumour pathogenesis and cell chemoresistance, as well as predicting the outcome of pharmacological treatments^16^,^17^,^20^,^21^,^22^. In particular, 3D Tissue Engineering (TE)-based *in vitro* models appear to be very promising. Although, so far, TE has focused primarily on regenerative medicine applications, it offers a potentially powerful toolbox for other areas in biomedical sciences: among these, the establishment of more physiologically reliable *in vitro* models^22^,^23^. TE cancer models can provide a number of advantages when compared to animal models, such as reproducibility, complexity (in terms of cell types, substrate chemistry, topography and mechanical properties, bioresorption, diffusion gradients, etc.) and ethical sustainability^17^.

These strategies aim to replicate the tissue/organ in culture, providing, in addition, those answers that cannot be solved using traditional approaches. For instance, we still have a very limited understanding of the nature of ECM signals decoded by mammary epithelial cells^24^. In order to carry out experimental investigations under physiological contexts, various 3D *in vitro* culture models have been proposed, with the aim of recreating cell-to-cell contact and the microenvironment surrounding cancer cells, as well as generating hypoxic-necrotic areas, therefore potentially contributing to tumour metabolism and progression and in metastasis formation^16^. Different types of scaffolds, ranging from non-woven fibre ECM-derived materials to polymers in the form of foams and hydrogels are being investigated^25^. Among these, natural or synthetic hydrogels offer several advantages such as good biocompatibility and bioactivity, high water content (which makes them similar to the native ECM), as well as efficient transportation of oxygen and nutrients due to the reticulated structure of cross-linked polymer chains^26^,^27^. Specifically, hydrogels have been used frequently for probing the microenvironment influence on cell functions, as their mechanical properties can be finely tuned in order to obtain stability in space and time^4^,^28^. Among them, seaweed-derived alginate is typically thought to be inert because it lacks the native bonds allowing interaction with mammalian cells^29^. For this reason, alginate allows the substrates’ mechanical contribution to cell fate the to be isolated better than chemically bioactive materials, such as Matrigel, laminin-rich or collagen matrices, which have already been adopted as 3D substrates for modelling cancer microenvironments^30^. Moreover, alginate mechanical properties can be precisely tuned via calcium ion mediated cross-links^31^,^32^.

In this study, we carried out a comparison to evaluate viability, proliferation rates and cluster organization in breast cancer cells (MCF-7) growing in mechanically tuned 3D alginate hydrogels. Among solid tumours, breast cancer has been chosen in this study, since satisfactory 3D *in vitro* models are not yet available although it remains the second leading cause of cancer death among women^33^.

We compared and quantified the differences detected in MCF-7 morphology and organization when cultured in environments of increasing complexity (i.e. standard plastic 2D environments, functionalized 2D environments and 3D gels), in order to highlight the need for more realistic 3D cancer models.

The evaluation of substrate stiffness effect on cell fate in a 3D environment takes into account cell mechano-transduction properties and lays the ground for the development of novel breast cancer *in vitro* models directly mediated by the mechanical properties of the environment.

## Results

**Figure 1:**
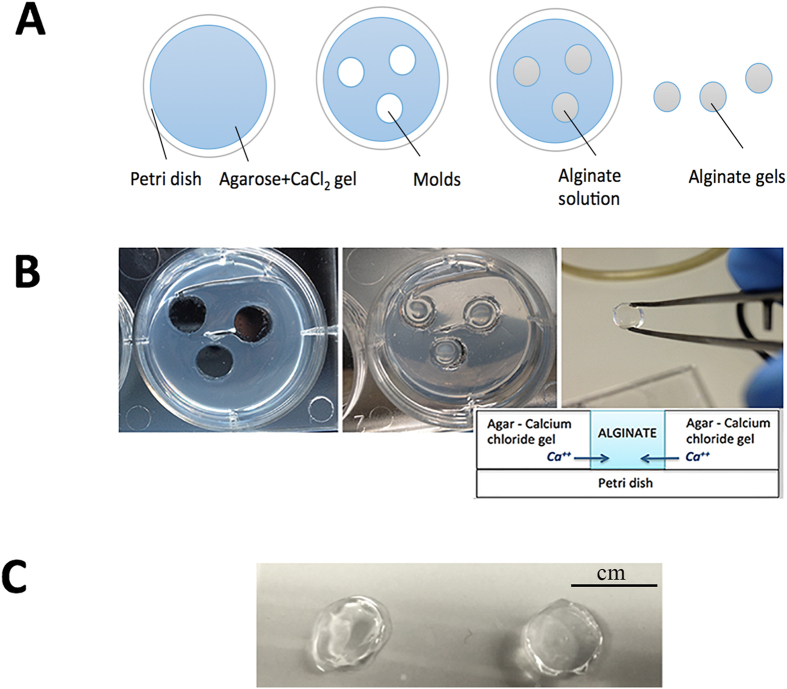
Gel manufacturing process. The protocol adopted for realization of the alginate gel consists of several steps, as summarized in the following picture: realization of the agar gel enriched with Calcium ions; realization of the gel molds, in the agar plates, using a Pasteur glass pipette; introduction of the alginate solution into the gel molds and alginate gelation. Panel A is a cartoon of these steps, while panel B shows some pictures of the experimental realization. Panel C shows two types of alginate gel samples prepared with 0.5% w/v (left) and 2% w/v (right) alginate solution and cross-linked with 0.5 M CaCl_2_ (scale bar: cm).

The adopted protocol produced 3D alginate gels, chemically cross-linked via radial diffusion of calcium ions (Fig. 1, panels A,B). Structural differences among gels produced with different cross-linker molarities and alginate concentrations were clearly observable: in detail, gels with 2% alginate solution and cross-linked with 0.5 M CaCl_2_ appeared structurally more compact and three-dimensionally defined than gels produced with either a lower concentration of alginate or a lower CaCl_2_ molarity (data not shown). In accordance with the dimensions of the gel molds, we obtained circular alginate gels, with ∼5 mm diameter and ∼3 mm height (Fig. 1, panel C).

### Gel mechanical characterization by AFM

The elastic modulus of each gel was measured at the sub-micrometer scale, the same length scale of the actual cell sensing^34^, using AFM nanoindentation technique.

**Figure 2:**
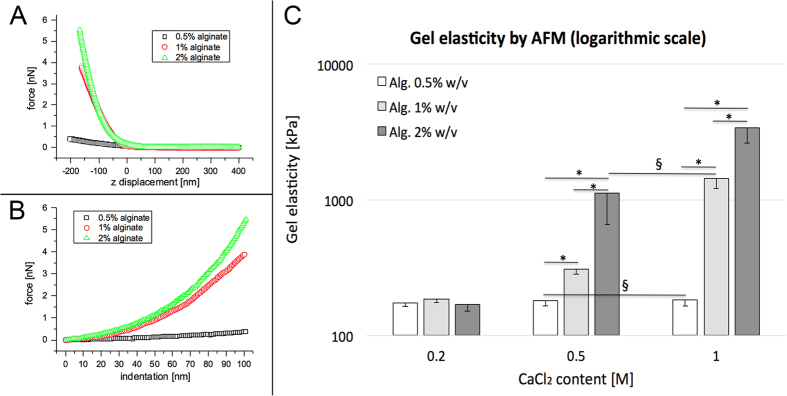
Gel mechanical characterization. Gel stiffness was measured by Atomic Force Microscopy. Panel A shows three representative force versus vertical displacement curves (average of 256 curves, full approach-retraction cycle is plotted) measured on three different gels (0.5% w/v, 1% w/v and 2% w/v alginate, all cross-linked with 1 M CaCl_2_). The z = 0 corresponds to the vertical piezo displacement where the AFM tip gets into contact with the gel surface. Panel B shows the plots of the corresponding load versus indentation curves; only the unloading portions are plotted. Panel C shows the elastic modulus (average and standard deviation, STD) for the different gels probed by AFM nanoindentation: bar colours correspond to different alginate concentrations, while calcium molarity is shown along the *x* axis. Symbol *indicates samples with statistically different elasticity (Mann-Whitney test, p < 0.05). Symbol ^§^indicates gels with elasticity ranges that are overlapped.

Figure 2 (panels A,B) shows, as an example, three representative indentation measurements for gels with 1 M CaCl_2_ and different alginate concentrations. The average force map (256 curves taken on a 5 × 5 μm^2^ area) of both the raw force-distance curves (panel A) and the corresponding load-indentation curves (panel B) are displayed. The latter were used to calculate the Young’s modulus of the gel. The curves show a qualitative, yet evident, difference in the compliance of the different gels while deformed by the AFM tip. Figure 2, panel C, reports the mean values and their dispersion of the Young’s modulus measured over the gel surface by AFM nanoindentation, following the procedure described in the methods section. As evidenced, a weaker cross-linker content (i.e. 0.2 M CaCl_2_) did not allow significantly different stiffnesses to be obtained among the gels, despite the increase in alginate percentage. On the contrary, the elastic moduli were significantly different at 0.5–1–2% alginate concentrations when gels were cross-linked with higher CaCl_2_ content (i.e. 0.5 M and 1 M). Gels with overlapping ranges of stiffness (i.e. 0.5% alginate-0.5 M CaCl_2_ with 0.5% alginate-1 M CaCl_2_ and 2% alginate-0.5 M CaCl_2_ with 1% alginate-1 M CaCl_2_) were merged as a unique range, to finally obtain four different categories of stiffness in which the gels were subdivided (i.e. 150–200 kPa; 300–350 kPa; 900–1800 kPa; 2500–4000 kPa).

Our results demonstrate that gel stiffness is highly dependent on CaCl_2_ cross-linker concentration. Table 1 reports some values from the literature^35^,^36^,^37^ on alginate gel elastic modulus, measured by AFM nanoindentation. Although CaCl_2_ concentrations do not overlap with our samples, the trend of increasing stiffness with an increase in both alginate and CaCl_2_ concentrations is in agreement with our observations.

### Cell viability

**Figure 3:**
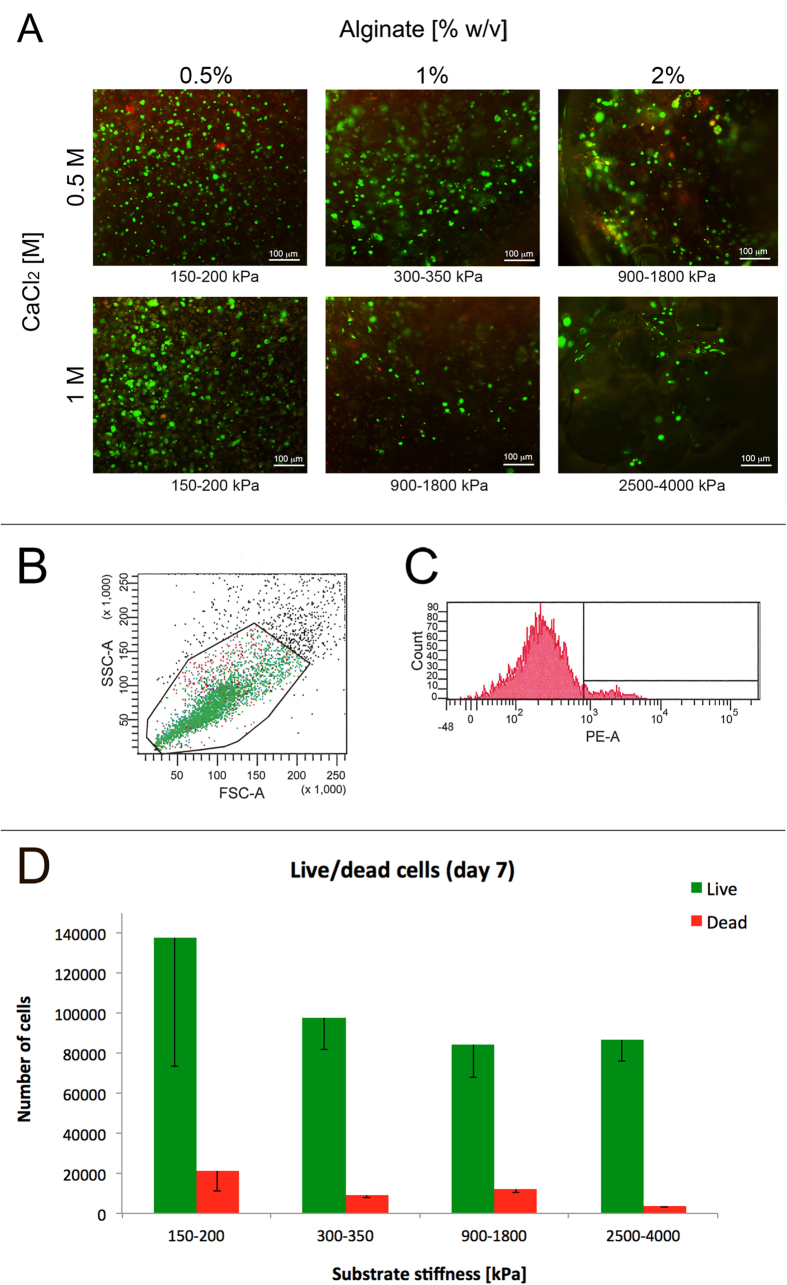
Cell viability. Representative images of live (green) and dead (red) MCF-7 cells encapsulated in alginate hydrogels with different alginate and CaCl_2_ concentrations, after 7 days of culture; images were acquired by fluorescence microscopy after treating samples with a Live/Dead assay; gels are co-labelled with their stiffness (panel A). Flow cytometric analysis of MCF-7 cells after 7 days of 3D culture in alginate gels. Viability of cells was analysed by setting a gate based on the Side- (SSC) and Forward- (FSC) light SCatter (panel B), and measuring the percentage of PI positive staining cells within the gate, indicating dead cells (panel C). Number of live and dead cells per sample type (samples with different stiffness), after 7 days, estimated by FACS analysis (panel D). Error bar represents Standard Deviation (STD).

Cell viability in the alginate gels was evaluated after 7 days of culture using both the Live/Dead cell assay and FACS analysis. In detail, we used a fluorescence-based Live/Dead assay to acquire qualitative information on cell vitality, while FACS analysis was used to obtain a semi-quantitative outcome on cell number within each gel type. From the images in Fig. 3, panel A, it is observable that only a few dead cells were found in gels after 7 days, confirming alginate as a promising substrate for cell culture and validating the adopted gelation protocol. However, a deep discrepancy in cell colonization among different gel types was evident.

For FACS analysis, MCF-7s were firstly characterized (Fig. 3, panels B and C) to set the best operational conditions; the number of live and dead cells was then derived for each gel type in relation to substrate stiffness (Fig. 3, panel D). FACS analysis confirmed and highlighted the differences in cell viability of the various gel types; in particular, the detected amount of cells is higher in gels characterized by a lower elastic modulus. The number of live cells seems to be influenced by substrate stiffness, while the number of dead cells (determined by propidium Iodide staining) is independent of substrate stiffness.

### Cell morphology: 2D versus 3D

Cell morphology and organization in 3D gels were analysed by observing cytoskeleton orientation and cell-to-cell contact using a confocal microscope. As the control, MCF-7 cells, seeded on both standard and alginate-coated Petri dishes, were observed.

**Figure 4:**
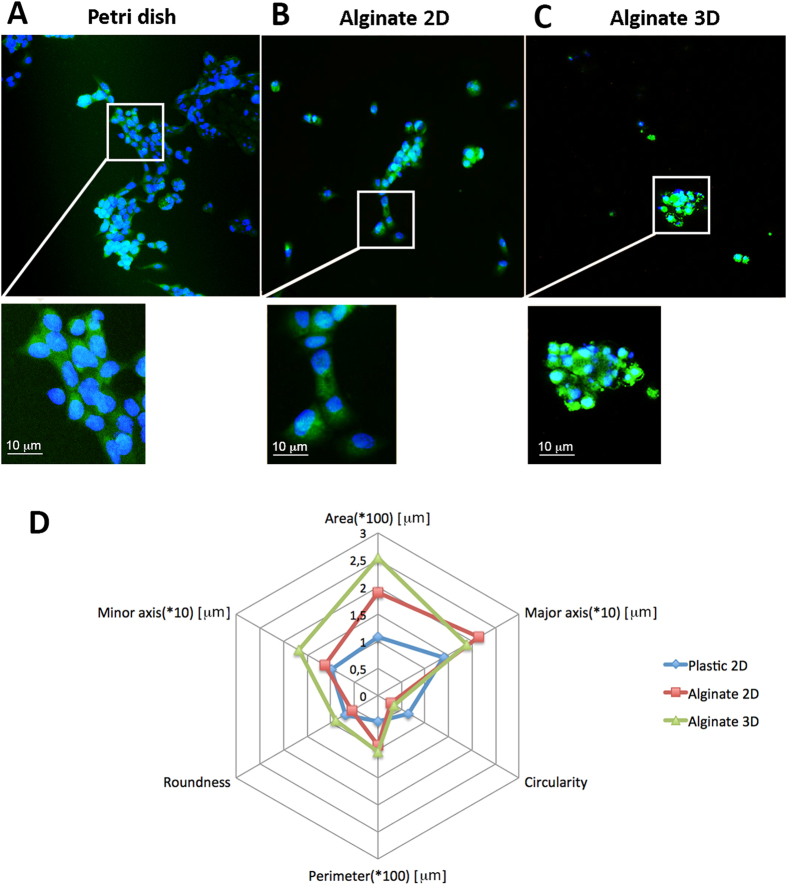
Cell morphology. Cells cultured on plastic (panel A), alginate-functionalized plastic (panel B) or embedded in 3D alginate hydrogels (panel C) show strongly different morphologies (blue: DAPI; green: Phalloidin). In particular, cells exhibit a flat shape in both 2D conditions; this morphology, even though self-explanatory of a good cell adhesion, heavily limits cell-to-cell contacts, which are maintained in 3D culture. Panel D shows quantitative information about cell morphologies in relation to their culture environment; in detail: area, perimeter, major axis, minor axis, circularity and roundness were analyzed for each culture condition. All the measures are expressed in μm, except Roundness and Circularity, which are dimensionless (values ranging from 0 to 1).

As expected^4^,^38^, the morphological observation after one week of culture showed alarming morphological differences between cells in 2D and 3D. In particular, cells showed a flat morphology and were organized in a monolayer when seeded in standard or alginate-coated Petri dishes (Fig. 4, panels A and B). In contrast, cells embedded within 3D gels were characterized by a round shape and cluster organization (Fig. 4, panel C). Several cell colonies were observed, and multiple nuclei were present in each colony meaning that each cluster consisted of many cells. These differences are of great impact considering that changes in morphology often go hand-in-hand with biochemical changes, as demonstrated by other scientists: in 2007, Kenny *et al*. not only showed that strong differences occurred in breast cancer cells when cultured in 2D or 3D, but classified several breast cancer cell lines with different aggressiveness, as proven by gene expression analysis, into four distinct morphological groups: Round, Mass, Grape-like and Stellate. In that study, MCF-7 cells belonged to the Mass group, since they formed colonies with disorganized nuclei and filled colony centres^39^.

Going beyond the current state-of-the-art, we measured the changes in MCF-7 morphological features when cultured in different dimensional configurations (2D or 3D). Our results show that cells in 3D are characterized by a greater Area and Perimeter than in 2D, and by a greater balance between Major and Minor Axes. Consequently, they show higher values of Roundness than cells in 2D, characterized by elongated shapes. Circularity values are not statistically different among the different conditions (Fig. 4, panel D).

### Cell cluster formation

**Figure 5:**
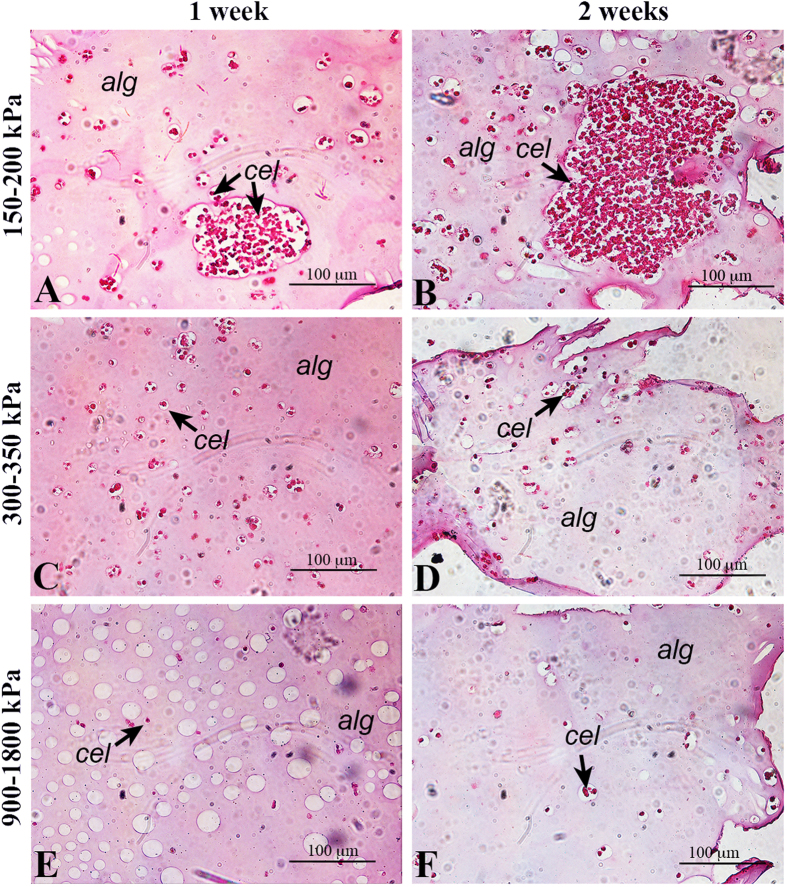
Cell proliferation and cluster formation. Histology of three alginate types (150–200 kPa, 300–350 kPa and 900–1800 kPa). Hematoxylin/eosin-stained sections are shown at 7 (left column) and 14 days (right column). Alginate (*alg*) is identified by the pink background, while cells (*cel*) are indicated by arrows and are purple. It is clearly observable that, if in stiff substrates cells are relatively isolated without significant proliferation rates both at 7 and 14 days (panels C, E and D, F, respectively), in soft gels they are highly proliferate, reaching clusters of 100 μm in one week (panel A) and 300 μm in two weeks (panel B).

The presence of cell clusters was investigated in 3D gels by histological analysis of the gel inlets. For this analysis, only gels which had given the best cell viability results (i.e. 150–200 kPa, 300–350 kPa and 900–1800 kPa stiffness) were analysed. Initially, cells were distributed evenly as single cells inside each type of gel (data not shown). After 7 days, most of the cells within the hydrogels with low stiffness (150–200 kPa) proliferated to form spheroids with a mean size of 100 μm (Fig. 5, panel A). In contrast, in stiffer gels, only a few cells composed the majority of clusters (Fig. 5, panels B and C). After 14 days, the size of aggregates in cell laden gels with low stiffness increased to 300 μm (Fig. 5, panel C); the cluster size remained almost constant in stiffer substrates (>300 kPa), where cells did not show similar proliferation ability (Fig. 5, panels D and E).

**Figure 6:**
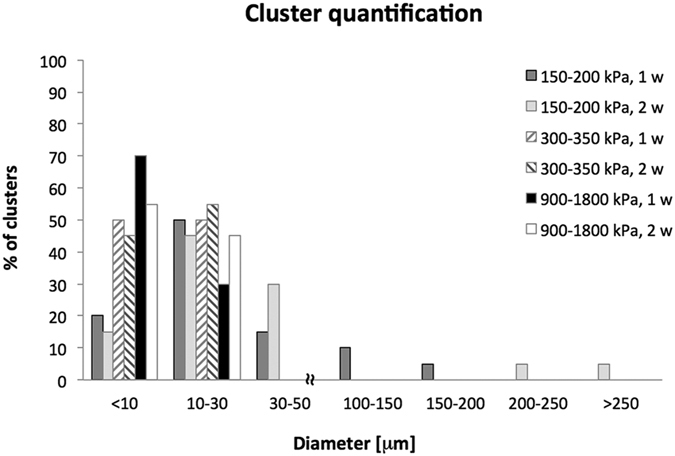
Cluster quantification. Occurrence (expressed as percentage) of cell clusters, subdivided by size, in gels with different stiffness. From the histogram it is observable that clusters greater than 30 μm are only found in the softest alginates (150–200 kPa). If their size is limited to a maximum of 200 μm when gels are cultured for 7 days, greater dimensions are found in the same gel types when cells are cultured for up to 14 days.

The presence of clusters in relation to their dimensions was analysed for each type of gel. Results are shown in Fig. 6. It can be observed that in moderately stiff (900–1800 kPa) and medium stiffness gels (300–350 kPa), clusters maintained small dimensions (<10 μm and about 30 μm, respectively) after both 1 and 2 weeks of culture. In contrast, in the softest gels (150–200 kPa), clusters reached greater dimensions (200 μm and >250 μm after 1 and 2 weeks of culture, respectively), and the percentage of isolated cells decreased.

In soft cell laden alginates, together with a higher cluster dimension, we also observed higher values of cluster density, which passed from 56% (±3,6%) during the first week to 70% (±6,5%) in the second week (Table 2).

## Discussion

The availability of 3D models offering the appropriate *in vitro* microenvironment for cell tumour growth is essential to improve our knowledge of cancer biology and successfully test new anticancer compounds.

Animal models have proven to be not entirely compatible with the human system, and the success rate between animal and human studies is still unsatisfactory. On the other hand, 2D cell cultures are useful tools for cancer studies but they fail to reproduce some crucial aspects of tumours, such as 3D cell growth and cell-matrix interactions. These limitations have a significant weight especially during the screening of novel drugs, since it has been demonstrated that cells become less sensitive to anti-cancer treatments when in contact with their microenvironment^40^.

Thus, to obtain the same cancer cell inhibition level observed *in vivo*, the culture environment has to reflect the 3D natural environment, including its mechanical cues. Indeed, the biochemical (e.g. adhesiveness) and physical properties (e.g. substrate stiffness) of the extracellular microenvironment have been recognized as independent factors that influence cell function and tissue morphogenesis in multiple ways^41^. Consequently, both categories must be considered when designing substrates for cell culture applications.

In this context, the field of tissue engineering (TE) offers powerful tools to achieve 3D *in vitro* cancer models that are representative of *in vivo* solid tumours. Some TE-based cancer models have already been proposed, showing promising results in recapitulating several aspects of tumour microenvironment complexity^16^. Above all, natural or synthetic hydrogels reported successful outcomes in mimicking the ECM environment, thanks to their high water content and remarkable biocompatibility. In particular, Collagen I-based hydrogels showed promising angiogenic potential with a significant up-regulation of vascular endothelial growth factor (VEGF) gene expression, representative of the pre-vascularized stages of *in vivo* solid tumour progression^42^. Laminin-rich gels were adopted and validated as 3D platforms able to distinguish non-malignant versus malignant breast cells: when embedded in such gels, the non-malignant cells were organized into polarized colonies similar to mammary acini, while malignant cells lost cell polarity and underwent the disorganized growth typical of *in vivo* tumours^43^. However, in these studies, the tentative of reproducing the chemical features of the native ECM made the investigation of cell response to mechanical stimuli (mechano-responses) more complicated^44^. In the current study, we have realized and characterized 3D alginate-based hydrogels. Although alginate has already been demonstrated to be an excellent substrate for breast cancer cell culture^45^, here we have stressed the physical properties of alginate gels, including their stiffness, with the final aim of studying the effect of substrate elasticity on breast cancer cell activity (i.e. viability, proliferation and cluster formation). Alginate serves as a model, since its properties reflect those of many other gels. The following considerations, however, can be applied to other polymers, being independent from the chemical properties of the substrates.

The first step of this work was dedicated to definition of the parameters (Alginate percentage and cross-linker CaCl_2_ content) capable of modulating gel stiffness, quantified as elastic modulus.

Among the techniques and laboratory instruments typically used for the mechanical characterization of 3D substrates, Atomic Force Microscopy (AFM) was chosen, since it allows measurement of hydrogel stiffness with a size-scale comparable to the cellular one, for a better understanding of the cell-substrate stiffness interaction.

Human breast cancer cells (MCF-7) were directly embedded within alginate gels with different elasticity, and cultured for up to 14 days. Cellular viability and cytoskeleton morphology were evaluated at early time points, since they are mostly a biological response to a specific event/environmental condition, e.g. substrate stiffness, chemical structure (plastic vs. alginate) and complexity (2D vs. 3D). Cell proliferation and cluster formation were analysed at both 7 and 14 days, in order to evaluate the progressive evolution of these processes within alginate-based gels.

**Figure 7:**
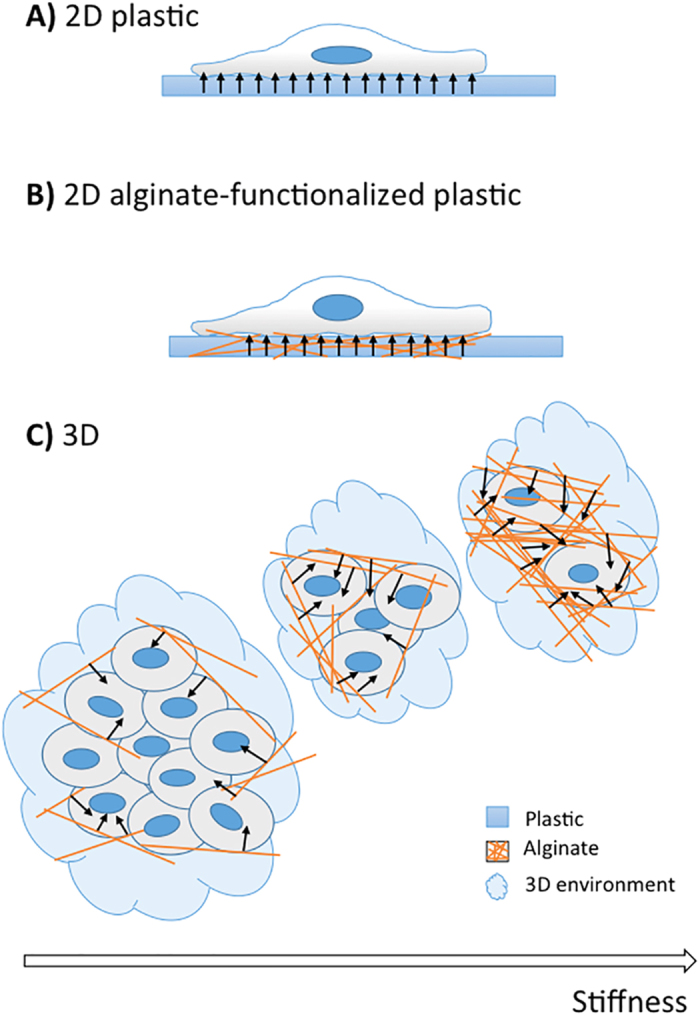
Schematic representation of the results. MCF-7 morphology and proliferation are deeply conditioned by microenvironment complexity (2D/3D) and matrix stiffness: cells maintain a flat morphology when cultured in standard 2D substrata (panel A) or in alginate coated-2D substrata (panel B), while they assume round morphology and organize in clusters when embedded in 3D gels (panel C). MCF-7 proliferation in 3D environments strictly depends on matrix stiffness (stiffness increasing is schematically represented by orange lines increasing): if the matrix is too rigid it exerts forces (arrows) that potentially inhibits cell proliferation.

Remarkably, our results show that stiffness directly influences cells fate, not only in a 2D culture set-up as already demonstrated^1^, but also in a more realistic 3D microenvironment where we observed a proportional elasticity-mediated cellular viability. In particular, we found that MCF-7s consistently proliferated in gels characterized by elastic moduli ranging between 150–200 kPa, until cell clusters of 100 μm- and 300 μm-diameters were obtained after 1 and 2 weeks, respectively. This uninhibited proliferative capacity, hereby observed in softer gels, is one of the key stages that identifies the initial pre-vascularization and growth of solid tumours^40^,^46^. Additionally, the multicellular, cluster-like, conformation observed in 3D gels was much closer to *in vivo* solid tumour organization than the one seen in both 2D Petri and 2D-alginate cultures, thus demonstrating the benefit of 3D cancer models for reproducing cell-cell and cell-matrix interactions^47^,^48^. As a consequence, we suggest that cellular morphology may be strongly affected by microenvironment dimensionality: tumour cells showed a flat morphology when expanded in 2D cultures, where only a segment of the cell membrane can interact with the substrate (Fig. 7, panels A,B), while they exhibited a round shape in the 3D environment. In this condition, their proliferation strictly depends on substrate stiffness, which may affect the diffusion of nutrients and intracellular signalling through a mechano-transduction mechanism (Fig. 7, panel C).

Although the proposed *in vitro* breast cancer model specifically addresses the evaluation of cellular response to different substrate elasticity ranges and avoids the overall complex nature of the tumour microenvironment, some key characteristics of breast tumours, such as cluster organization and cellular morphology, have been successfully reproduced here. Therefore, it is desirable and expected that such a model could be enriched with further features (e.g. chemical and biological functionalization) to better emulate *in vitro* the natural tumour niche. These advanced models could be finally used for initial therapeutic screenings, e.g. evaluating if the administration of anticancer agents causes cell apoptosis or breaks cell proliferation as observed in successful *in vivo* chemotherapy treatments.

## Methods

### 3D alginate gel preparation

Three-dimensional alginate gels were prepared as follows. Firstly, 1% (w/v) agar solutions containing calcium ions (*solutions A*) were prepared by mixing Agar (DIFCO Laboratories) in physiological buffer enriched with different concentrations (0.2 M, 0.5 M or 1 M) of CaCl_2_ (J. T. Baker). The solutions were brought to the boil, poured into 6-well plates until ∼1 cm height was obtained and allowed to cool until complete solidification. Holes of 0.5 cm diameter were then cut in the agar, using a Pasteur glass pipette, to make gel molds. Alginate solutions, 0.5% (w/v), 1% (w/v) or 2% (w/v), were prepared by mixing Alginate (Manugel GMB, FMC BioPolymer) in physiological buffer (*solutions B*). Effective intimate mixing of the alginate solutions was carried out for 12 h at room temperature under vigorous magnetic stirring. The solutions were then stored at 4 °C.

Next, 120 μm of each solution B was dispensed into the selected gel molds using a syringe. Gelation was allowed to take place at 37 °C for 1 hour, in order to ensure complete diffusion of calcium ions from the agar to the alginate solution. The alginate hydrogels were then extracted from the gel molds and maintained in a buffer containing 5 mM CaCl_2_.

### Atomic Force Microscopy (AFM) nanoindention

Atomic force microscopy (AFM) was used to measure gel stiffness. To do this, we used a commercial AFM microscope (Keysight Technologies 5500 ILM) equipped with a closed loop scanner capable of 9 *μm* vertical range. The scanner was always operated in “closed loop” in order to compensate for piezo nonlinearity, creep and hysteresis. A rectangular, 250 μm long, silicon microcantilever with sharp conical tips and a cone angle of 22° was used (CSG 11 type, NT-MDT, Russia). The cantilever spring constant was calculated by monitoring the cantilever oscillation in air due to thermal noise, following the procedure described by Hutter and Bechhoefer^49^.

Gels were glued onto a Petri dish using a minimum amount of fast cyanoacrylate glue. During the gluing step, the specimens stayed less than a minute out of the solution^50^. During measurement, samples were kept in a buffer containing 5 mM CaCl_2_.

AFM indentation measurements were performed by recording a standard force curve and then calculating the corresponding load versus indentation curve.

The applied load for any given cantilever deflection was calculated by first converting the output voltage, from the AFM four-segment photodetector, into nanometers of deflection (sometime referred to as “inverse optical lever sensitivity”), and then by multiplying the deflection by the cantilever spring constant. The conversion factor was calculated by taking several force curves on a hard glass substrate, every time the laser spot on the cantilever had to be adjusted, and considering the reciprocal of the average slope of the constant compliance region of the curves.

In order to take into account intra-sample heterogeneity, 16 × 16 = 256 force curves were recorded over a regular grid over a 5 × 5 μm Each force curve was taken at a constant vertical displacement speed of 6 μm/s and with a maximum applied load that varied from sample to sample in order to get a maximum sample deformation (i.e. indentation) of 100 nm.

Single force curves were processed in a semi-automatic way using a custom built software in order to (i) detect the vertical displacement corresponding to the AFM probe-gel surface contact (during this procedure 10% of the curves on average were discarded due to noise or artefacts during acquisition which prevented the detection of the point of contact), (ii) extract the applied load *P* as a function of the sample indentation *h* (the so-called load-indentation curve).

In order to calculate the Young’s modulus of the gel we considered the unloading portion of the load-indentation curve to avoid effects due to plastic deformations and referred to the model for a quasi-static indentation with a conical indenter originally proposed by Oliver and Pharr^51^ and then refined by the same authors^52^:


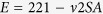


where 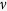 is the Poisson’s ratio of the hydrogel, ***A*** is the projected indenter contact area at a given indentation, and ***S*** the contact stiffness, defined as the derivative of the load-indentation curve at the same indentation. We calculated, for all measurements, contact area and stiffness at the maximum indentation 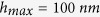. Applying the same model, in order to calculate the derivative at 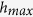, we fitted the load-indentation relation with a power law: 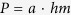 where 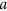 and *m* are the fitting parameters (*m* lays in the range 1.5–2 for a conical indenter^52^).

We assumed, for the Poisson’s ratio of all tested gels, a constant value of 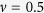, corresponding to an uncompressible, rubber like, material.

On a single gel sample, at least three maps of 16 × 16 curves over grouped collected onto macroscopically different positions randomly selected over the sample surface. Hence the reported elasticity value for each gel type corresponds to the average of at least 690 single measurements and evaluations of the Young’s modulus.

Statistical evaluation was performed using the non-parametric Mann-Whitney test to determine significant differences among groups. The significance level was set at p < 0.05. All results are presented as mean ± standard deviation.

It should be noted that the indentation measurements were not performed in quasi-static conditions and therefore the viscous response (loss modulus) might play a significant role; moreover the calculated absolute values of elasticity are prone to several uncertainties and non-idealities (e.g. in the tip geometry or the gel Poisson’s ratio). Nevertheless, since all AFM measurements reported in this paper were taken with the same cantilever and the same experimental conditions, the observed relative changes in stiffness are not significantly affected by the above uncertainties.

### MCF-7 cell culture

The MCF-7 (breast adenocarcinoma) cell line was obtained from Sigma Aldrich. Cells were expanded in Eagle’s minimal essential medium (EMEM) enriched with 10% Fetal Bovine Serum (FBS), 1% L-glutamine and 1% penicillin/streptomycin (all from Sigma Aldrich). When the required confluence was reached, cells were detached with 1X trypsin and counted. Cell seeding within the gels was achieved by directly suspending cells in sterile alginate solution in order to obtain an inoculum of 10^5^ cells/sample. Culture medium enriched with 5 mM CaCl_2_ was then added. Two-dimensional cell cultures were carried out as a control to evaluate whether differences occurred on cells when cultured in 2D or 3D conditions. To this aim, MCF-7 cells were seeded at 3500 cells/cm^2^ density on both standard plastic and alginate-coated Petri dishes.

Both 2D and 3D cell cultures were incubated for up to 2 weeks at 37 °C in an atmosphere of 5% CO_2_ to allow gas exchange. Medium was changed twice a week. At least duplicate samples were used for each assessment.

### Live/Dead staining

After 7 days, cellular viability was analyzed in the 3D gels. For this purpose, gels were washed with phosphate-buffered saline (PBS) and incubated with a Live/Dead stain (Live/Dead Cell Double Staining Kit, Sigma Aldrich) at 37 °C for 15 minutes.

Gels were then imaged by using an upright microscope equipped with transmitted illumination and epifluorescence (Eclipse Ni-U, Nikon)^53^ to discriminate live cells (calcein AM stained-green) from dead cells (propidium iodide stained-red).

### Flow cytometry

After 1 week, at least three gels per type were de-cross-linked by incubating them for 2 hours with a 1:1 (v/v) solution of 50 mM EDTA and complete medium. Cells were suspended in PBS enriched with 2% FBS (v/v) and stained for 5 minutes by incubating in Propidium Iodide (PI) solution (1% v/v). Flow cytometry was performed on a FACSCalibur system to obtain total and dead cell (PI-stained) counts.

### Confocal fluorescence microscopy and morphological analysis

To examine the spatial distribution and morphology of cells, both 2D and 3D samples were analysed by optical confocal laser-scanning microscopy. For all typologies, samples were fixed, after 1 week, with 4% paraformaldehyde and treated with 0.1% Triton X-100 to permeabilize the cell membrane. Nuclei were stained with 1 μg/ml 4,6-Diamidino-2-phenylindole (DAPI), while actin filaments were stained with 100 μM Alexa Fluor 488 Phalloidin (all Sigma-Aldrich). Images were acquired by a confocal laser-scanning microscope (Leica TCS SP5 AOBS). Alexa Fluor 488 was excited with the 488 nm line of the Ar laser and its fluorescence was collected in a spectral window of 500 to 530 nm. For DAPI, 720 nm excitation wavelength and 450–500 nm spectral window emission were used.

Images were then analysed by ImageJ to measure and quantify several morphological features characterizing cells grown both in 3D and in 2D conditions. In particular, the following parameters were considered: Area of the cell; Perimeter of the cell; Major Axis and Minor Axis of the best fitting ellipse; Circularity, defined as 4πArea/Perimeter^2^; Roundness, defined as Minor Axis/Major Axis.

### Histology and cluster analysis

After 1 and 2 weeks of culture, the 3D alginate hydrogels were processed for histological analysis in order to observe the time-dependent evolution of cell clusters. Briefly, samples were fixed in 4% buffered formalin for 3 hours and dehydrated in ethanol scale for a total of 6 hours. The samples were then paraffin embedded, cross-sectioned (7-μm thick) at different levels and stained with haematoxylin–eosin (H&E)^54^,^55^. Images were acquired by using a Nikon H550L optical microscope.

We evaluated two parameters of cell clusters: dimension and density.

For dimension analysis, at least 10 clusters (intended as group of communicating cells) were considered for each hydrogel, and the major axis of each cluster (i.e. cluster size) was measured. These values were subdivided into ranges (i.e. <10 μm, 10–30 μm, 30–50 μm, 100–150 μm, 150–200 μm, 200–250 μm, >250 μm) and clusters classified; results are presented as percentage of occurrence (Fig. 6).

Cluster density, defined as 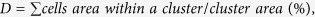 was quantified (Table 2). To this aim, images of clusters were post-processed by using Image J platform: they were converted to 8-bit images and an automatic threshold was applied to discriminate the space occupied by cells (black) from the background (white). Threshold accuracy was checked manually for each image. Cluster density was calculated, in percentage, as ratio between black space and the total area of the cluster.

## Additional Information

**How to cite this article**: Cavo, M. *et al*. Microenvironment complexity and matrix stiffness regulate breast cancer cell activity in a 3D in vitro model. *Sci. Rep.* **6**, 35367; doi: 10.1038/srep35367 (2016).

## Figures and Tables

**Table 1:** Values from the literature of alginate gel elastic modulus, measured by AFM nanoindentation.

Alginate	0.01 M	0.06 M	0.1 M
CaCl_2_			
0.7%			3.2 kPa^35^
1%			3.6 kPa^36^
1.5%			9 kPa^35^
2%			6 kPa^36^
3%			19 kPa^35^
5%	4.77 kPa^37^	56.98 kPa^37^	60.60 kPa^37^

**Table 2:** Cluster density calculated as the ratio between the space occupied by cells and the size of the cluster.

	1 week	2 weeks
Cluster density (cells/area %)	56,3 (±3,62) %	70,75 (±6,5) %

This value increases from the first to the second week of culture, meaning that clusters are more densely packed with cells.
